# Esophageal strictures associated with prolonged nasoesophageal tube placement in three cats

**DOI:** 10.1093/jvimsj/aalag289

**Published:** 2026-11-10

**Authors:** Jaechun Cho, Minsuk Kim, Sehun Kim, Jeongmin Lee, Hyung-Kyu Chae, Chul Park

**Affiliations:** Veterinary Internal Medicine, Jeonbuk National University, Iksan, Republic of Korea; Veterinary Internal Medicine, Jeonbuk National University, Iksan, Republic of Korea; May Animal Medical Center, Jeonju, Republic of Korea; Cheongju Korea Animal Medical Center, Cheongju, Republic of Korea; College of Veterinary Medicine, Kyungpook National University, Daegu, Republic of Korea; Veterinary Internal Medicine, Jeonbuk National University, Iksan, Republic of Korea

**Keywords:** enteral nutrition, feeding tube, mucosal injury, regurgitation

## Abstract

Nasoesophageal tube placement for a prolonged period of time has not previously been associated with esophageal stricture in cats. Three cats with suspected hepatic lipidosis required extended enteral nutritional support because voluntary food intake remained < 25% of the calculated resting energy requirement. The cumulative duration of nasoesophageal tube placement was 24-37 days. Regurgitation or retching developed within 1-3 days after tube removal, and esophagoscopy identified severe focal strictures in the cervical esophagus at the C4-C6 vertebral level in all 3 cats. One cat underwent 3 endoscopic balloon dilatation sessions at 1-week intervals, with enlargement of the esophageal lumen. The consistent anatomic location and temporal association with prolonged tube placement suggest that extended nasoesophageal intubation may be a risk factor for cervical esophageal stricture formation in cats. When prolonged nutritional support is anticipated, transition to a feeding tube intended for long-term use should be considered.

## Introduction

Nasoesophageal and nasogastric tubes are commonly used to provide temporary enteral nutrition in dogs and cats when voluntary food intake is inadequate, with nasoesophageal tubes typically used for fewer than 4-5 days.^1–3^ These tubes require minimal sedation, can be placed rapidly, and permit administration of liquid diets and medications.^4–6^ Despite these practical advantages, nasoesophageal or nasogastric tube placement and maintenance are associated with several complications.

Reported complications of nasoesophageal or nasogastric tube use in veterinary patients include vomiting, regurgitation, tube obstruction or displacement, nasal irritation, aspiration, and inadvertent placement into the respiratory tract.^7–10^ Most available veterinary information concerns relatively brief periods of tube placement, and esophageal stricture has not been well characterized as a complication of extended nasoesophageal or nasogastric tube use in cats. Esophageal injury and subsequent stricture formation in cats have also been associated with oral administration of certain medications, particularly doxycycline.^11^

In people, esophageal stricture is an uncommon but recognized complication of prolonged nasogastric intubation. Proposed mechanisms include gastroesophageal reflux, impaired esophageal clearance, and direct mechanical injury to the esophageal mucosa.^12^^,^^13^

We describe development of cervical esophageal strictures in 3 cats after prolonged nasoesophageal tube placement.

## Cases

### Case 1

A 6-year-old, 4.5-kg, neutered male Persian cat with a body condition score of 4/9 was presented for a 7-day history of anorexia and lethargy. On physical examination, marked generalized icterus involving the skin, sclera, and oral mucous membranes was present. Serum biochemistry abnormalities included marked hyperbilirubinemia (total bilirubin, 11.5 mg/dL; reference interval [RI], 0.1-0.4 mg/dL) and increased activities of alkaline phosphatase (2728 U/L; RI, 38-165 U/L), γ-glutamyl transferase (16 U/L; RI, 1-10 U/L), alanine aminotransferase (622 U/L; RI, 22-84 U/L), and aspartate aminotransferase (197 U/L; RI, 18-51 U/L). Abdominal ultrasonography identified hepatomegaly with diffusely increased hepatic echogenicity and gallbladder distension. Based on prolonged anorexia, marked hyperbilirubinemia, and the ultrasonographic findings, hepatic lipidosis was suspected. Fine needle aspiration of the liver was not performed because the diagnosis was considered clinically likely, and enteral nutritional support was prioritized because voluntary food intake remained < 25% of the calculated resting energy requirement.

The cat was hospitalized and received IV fluid therapy, maropitant (1 mg/kg IV q24h), esomeprazole (1 mg/kg IV q24h), silymarin, and S-adenosylmethionine (10 mg/kg PO q12h). A 6-Fr, 50-cm, di(2-ethylhexyl) phthalate (DEHP)-free feeding tube (F.D.T[P], JMS Co., Ltd., Seoul, Republic of Korea) was measured from the nostril to the distal thoracic esophagus, lubricated, advanced through the ventral nasal meatus, and secured at the nostril. Correct positioning of the nasoesophageal tube within the distal thoracic esophagus was assessed by a negative-pressure check after insertion and subsequently confirmed on thoracic radiographs without administration of contrast medium before feeding (Figure S1). The cat remained hospitalized throughout the period of enteral nutritional support. Enteral feeding was initiated at 25% of the calculated resting energy requirement. By day 5, enteral feeding had been increased to 100% of the calculated resting energy requirement, and body weight was 4.45 kg. Although the cat’s activity and hepatobiliary biochemical abnormalities gradually improved, voluntary food intake remained inadequate to meet its calculated resting energy requirement. Approximately 10 days after initial tube placement, the nasoesophageal tube was temporarily removed to assess voluntary food intake, but the cat failed to consume an adequate amount of food, and a new nasoesophageal tube was placed the next day. Reinsertion was achieved on the first attempt without noticeable resistance. Nasoesophageal tube feeding therefore was continued. The cumulative duration of nasoesophageal tube placement was 37 days, with final removal on day 38. On day 39, the serum total bilirubin concentration had decreased to 0.6 mg/dL.

On day 39, 1 day after tube removal, the cat developed recurrent regurgitation and retching, particularly after eating. These signs persisted despite treatment with esomeprazole (1 mg/kg IV q24h), sucralfate (0.25 g/cat PO q8h), and metoclopramide (0.3 mg/kg IV q12h). Esophagoscopy performed 3 days after onset of clinical signs, on day 42, identified a severe focal circumferential stricture in the cervical esophagus at the fourth to sixth cervical vertebral (C4-C6) level, with an estimated luminal diameter of approximately 2-2.5 mm and focal mucosal hemorrhage within irregular fibrotic-appearing tissue (Figure 1A). The previously described medications were continued after the diagnosis of esophageal stricture. Endoscopic balloon dilatation was discussed with the owner but was not immediately pursued. On day 44, 2 days after the diagnosis of esophageal stricture, the cat developed acute respiratory distress after repeated episodes of regurgitation. Thoracic radiographs showed severe multifocal pulmonary infiltrates consistent with aspiration pneumonia. Given the severity of the respiratory compromise and poor prognosis, the owner elected euthanasia.

**Figure 1:**
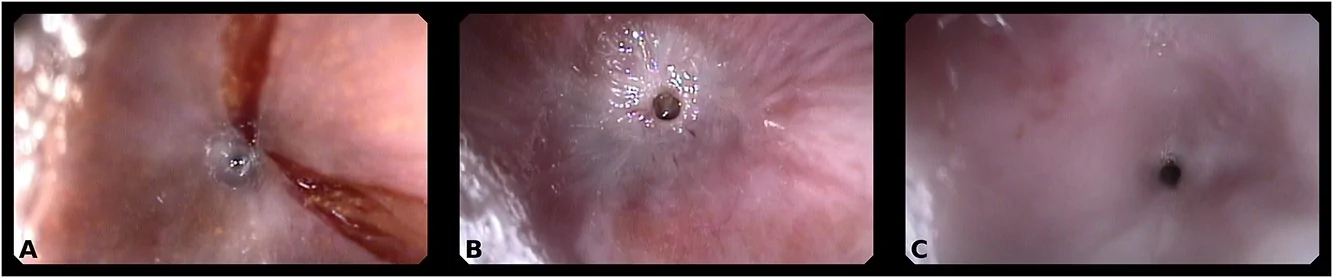
Endoscopic appearance of cervical esophageal strictures in 3 cats after prolonged nasoesophageal tube placement. (A) Case 1, showing a severe focal circumferential stricture at the C4-C6 level with marked luminal narrowing and irregular fibrotic-appearing mucosa. (B) Case 2, showing a severe focal cervical esophageal stricture at the C4-C6 level with concentric luminal narrowing, pale fibrotic-appearing tissue, and focal mucosal erythema. (C) Case 3, showing a severe focal circumferential stricture at approximately the C4-C6 level with marked residual luminal narrowing.

### Case 2

A 2-year-old, 4.8-kg, spayed female Korean Shorthair cat with a body condition score of 4/9 was presented for a 5-day history of lethargy, anorexia, and intermittent vomiting. Physical examination identified generalized icterus. Serum biochemistry abnormalities, measured using the same analyzer and reference intervals as in case 1, included hyperbilirubinemia (total bilirubin, 6.9 mg/dL) and increased activities of alkaline phosphatase (1107 U/L), alanine aminotransferase (369 U/L), and aspartate aminotransferase (111 U/L); γ-glutamyl transferase activity was within the reference interval. Abdominal ultrasonography showed hepatomegaly with diffusely increased hepatic echogenicity. Hepatic lipidosis was suspected. For the same reasons described in case 1, fine needle aspiration of the liver was not performed, and enteral nutritional support was initiated because voluntary food intake remained < 20% of the calculated resting energy requirement.

The cat received supportive care consisting of IV fluid therapy, maropitant (1 mg/kg IV q24h), esomeprazole (0.5 mg/kg IV q12h), silymarin, and S-adenosylmethionine (10 mg/kg PO q12h). On day 2, a 6-Fr nasoesophageal feeding tube was placed as described for case 1, and correct positioning was confirmed radiographically before feeding. Enteral nutrition was gradually increased according to tolerance. By day 5, enteral feeding had been increased to 100% of the calculated resting energy requirement, and body weight was 4.86 kg. Although the cat’s clinical condition and hepatobiliary biochemical abnormalities improved, voluntary food intake remained inadequate.

Fourteen days after initial tube placement, the nasoesophageal tube was removed to assess voluntary food intake. Because the cat failed to consume an adequate amount of food, it was returned the next day, and a new nasoesophageal tube was placed for continued nutritional support. The cat was admitted again for hospitalization after tube replacement.

Nasoesophageal tube feeding was subsequently continued, with final tube removal on day 27. By day 29, the serum total bilirubin concentration had decreased to 0.5 mg/dL.

On day 30, 3 days after tube removal, the cat developed intermittent regurgitation immediately after eating. Because the episodes were intermittent and the owner had concerns regarding the cost and anesthesia required for esophagoscopy, conservative management with sucralfate (0.25 g/cat PO q8h) and metoclopramide (0.3 mg/kg PO q12h) initially was pursued. Esophagoscopy was subsequently performed 18 days after onset of clinical signs, on day 48, and identified a severe focal cervical esophageal stricture at the C4-C6 level, with an estimated luminal diameter of approximately 3-3.5 mm, pale fibrotic-appearing tissue, and focal mucosal erythema (Figure 1B). Endoscopic balloon dilatation and placement of an esophagostomy tube were discussed with the owner but were not pursued because of financial constraints. A smaller 5-Fr nasoesophageal feeding tube was subsequently placed for nutritional support, and the cat was discharged for home care. Further follow-up information was unavailable.

### Case 3

An 8-year-old, 4.5-kg, spayed female Korean Shorthair cat with a body condition score of 5/9 was presented for a 6-day history of decreased appetite, lethargy, and intermittent vomiting. Physical examination identified mild generalized icterus and dehydration. Serum biochemistry abnormalities, measured using the same analyzer and reference intervals as in case 1, included hyperbilirubinemia (total bilirubin, 6.5 mg/dL) and increased activities of alkaline phosphatase (2037 U/L), alanine aminotransferase (236 U/L), and aspartate aminotransferase (188 U/L); γ-glutamyl transferase activity was within the reference interval. Abdominal ultrasonography identified diffusely increased hepatic echogenicity. The overall findings were considered most consistent with hepatic lipidosis. For the same reasons described in case 1, fine needle aspiration of the liver was not performed, and enteral nutritional support was prioritized.

Supportive management was the same as in case 1. Because voluntary food intake remained < 20% of the calculated resting energy requirement despite assisted feeding, a 6-Fr nasoesophageal feeding tube was placed on day 3 as described earlier, and correct esophageal positioning was confirmed radiographically before feeding. Enteral feeding was initiated at 30% of the calculated resting energy requirement and gradually increased as tolerated. Four days after tube placement, enteral feeding had been increased to 100% of the calculated resting energy requirement, and body weight was 4.4 kg. Despite improvement in its clinical condition and hepatobiliary biochemical abnormalities, voluntary food intake remained inadequate, and the tube was maintained for nutritional support. Ten days after initial tube placement, the nasoesophageal tube was removed, and the cat was discharged to assess voluntary food intake at home. Because the cat failed to consume an adequate amount of food, it was returned the next day, and a new nasoesophageal tube was placed for continued nutritional support. The cat was subsequently admitted again for hospitalization, and nasoesophageal tube feeding was continued. By day 27, the serum total bilirubin concentration had returned to within the reference interval.

On day 43, 2 days after final tube removal, the cat developed recurrent retching and regurgitation after eating. Esophagoscopy performed 5 days after onset of clinical signs, on day 48, identified a severe focal circumferential stricture in the cervical esophagus at approximately the C4-C6 level (Figure 1C). The narrowed lumen was estimated to be 2.5-3 mm in diameter, and the surrounding mucosa appeared pale pink without an obvious mass lesion, consistent with a benign fibrous stricture. Lateral contrast esophagography and fluoroscopic examination confirmed focal narrowing of the cervical esophagus at the same level (Figure 2A and B).

**Figure 2:**
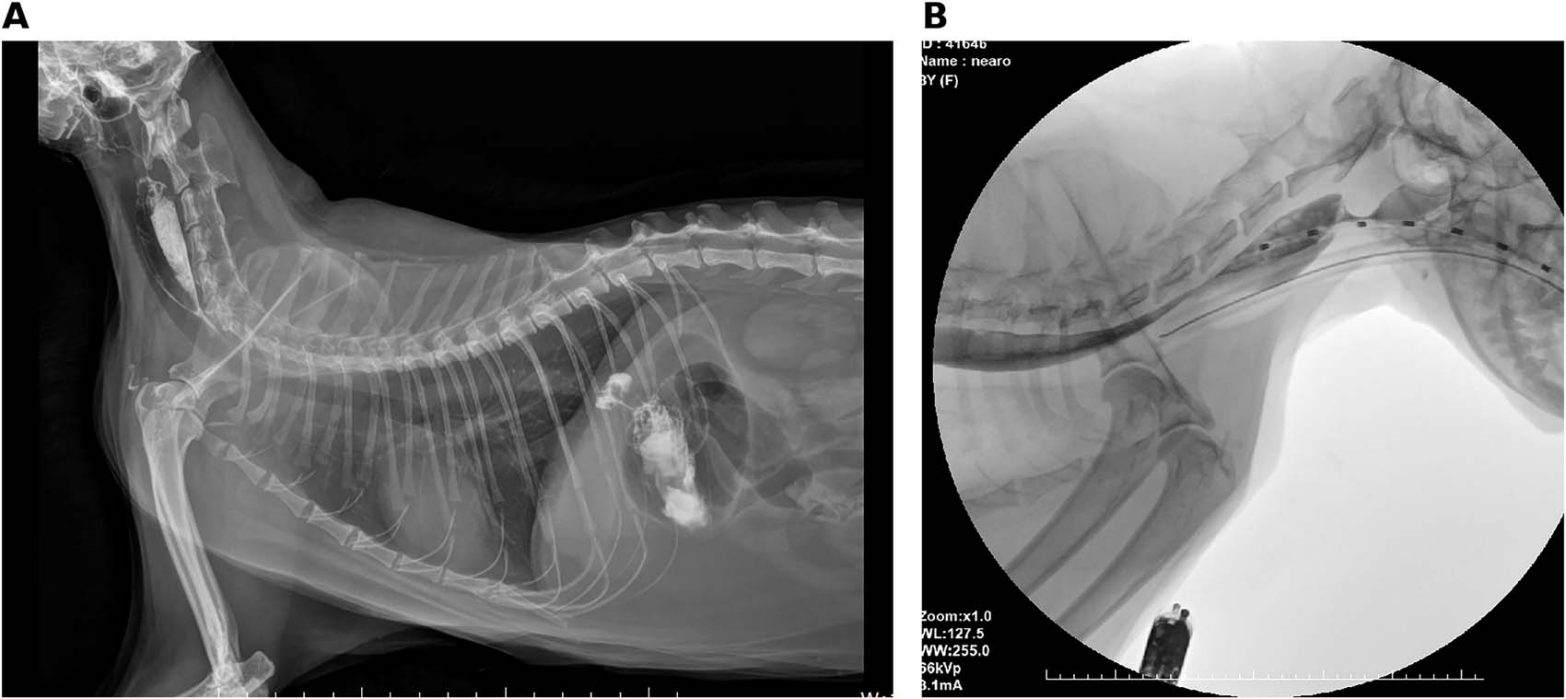
Radiographic and fluoroscopic confirmation of the cervical esophageal stricture in case 3. (A) Lateral contrast esophagogram demonstrating focal narrowing of the cervical esophagus with retention of contrast medium proximal to the stenotic segment. (B) Lateral fluoroscopic image confirming focal narrowing of the cervical esophageal lumen at the same level.

The cat underwent 3 balloon dilatation procedures, each performed 1 week apart, using CRE balloon dilatation catheters (Boston Scientific, Marlborough, MA, USA). During the initial procedure, the stricture was sequentially dilated using 6-mm and 10-mm balloons under combined esophagoscopic and fluoroscopic guidance, with the final inflation maintained for 1 min. Postprocedural management included sucralfate, esomeprazole, and a softened diet. After the final dilatation, repeat esophagoscopy confirmed sufficient luminal enlargement for endoscope passage. The cat was discharged on sucralfate and esomeprazole for 1 week and resumed voluntary food intake. At 3 months, regurgitation had not recurred and body weight had increased by approximately 15%.

## Discussion

We describe 3 cats with focal cervical esophageal strictures suspected to be associated with prolonged nasoesophageal tube placement. The cumulative duration of nasoesophageal tube placement was 24-37 days, and regurgitation developed within 1-3 days after final tube removal. Esophagoscopy identified severe focal circumferential strictures at the C4-C6 level in all 3 cats. Cervical esophageal strictures were uncommon in previous veterinary series, accounting for 22% and 10% of reported strictures, respectively, whereas most involved the middle or distal esophagus.^14^^,^^15^ To our knowledge, esophageal stricture associated with prolonged nasoesophageal tube placement has not previously been reported in cats. The consistency in tube exposure, anatomic location, and temporal onset of clinical signs in these cases suggests a possible association between prolonged nasoesophageal intubation and cervical esophageal stricture formation. Other reported risk factors include medication-associated esophageal injury, particularly with PO doxycycline; none of the cats had received doxycycline.^11^ All 3 cats also underwent tube removal and replacement, which may have caused additional mechanical trauma.

In the cats described here, enteral feeding was required because voluntary food intake remained insufficient despite improvement in suspected hepatic lipidosis. Attempts to discontinue tube feeding were made during hospitalization or after discharge, but inadequate voluntary intake necessitated tube replacement and continued nutritional support. When prolonged enteral support is anticipated, esophagostomy or gastrostomy tubes generally are preferred because they are more suitable for longer-term feeding.^1^^,^^16^ However, in some cases, conversion to an alternative feeding tube may be limited by factors such as the need for anesthesia, financial considerations, or owner preference. Accordingly, transition to a feeding tube intended for longer-term use should be considered when prolonged nutritional support is anticipated rather than extending the time of nasoesophageal tube use.

Previous veterinary studies have evaluated complications associated with nasoesophageal and nasogastric feeding tubes. In a prospective study of 97 dogs and cats, placement complications occurred in approximately one-quarter of patients and included sneezing, failed advancement, patient movement, oropharyngeal curling, discomfort or intolerance, coughing, nasal bleeding, and cardiopulmonary or respiratory arrest; tube misplacement in the respiratory tract occurred in 3.1%. During tube use, complications occurred in approximately half of patients and included sneezing, regurgitation, attempted self-removal, obstruction, nasal discharge, and coughing.^7^ In the previous report, the median indwelling duration was 2 days, with a maximum of 11 days, and esophageal stricture was not reported.^7^ In a retrospective study of 46 dogs, vomiting or regurgitation occurred in 11%, diarrhea in 9%, and tube displacement in 7%, whereas aspiration pneumonia and tube obstruction were uncommon. The median feeding duration was 3 days, and no esophageal strictures were identified.^8^ In contrast, each cat underwent 2 periods of nasoesophageal tube placement separated by a brief interval of tube removal, resulting in a cumulative tube placement duration of 24-37 days.

Esophageal stricture is a rare complication of nasogastric tube use in people. In one series of 26 patients, tube placement ranged from 2 to 90 days (mean, 19 days).^12^ Another report described a severe esophageal stricture after prolonged nasogastric intubation that required repeated balloon dilatation.^13^ These findings suggest that prolonged contact between the feeding tube and esophageal wall may contribute to clinically relevant mucosal injury, although the duration required for stricture formation is variable.

The mechanism of stricture formation in the cats described here remains uncertain. Nasoesophageal and nasogastric tubes may interfere with normal gastric reflux preventative mechanisms, impair lower esophageal sphincter closure and esophageal clearance, and thereby increase exposure of the esophageal mucosa to refluxate.^12^^,^^17^ However, reflux injury alone would not readily explain the consistent cervical distribution observed in our cases. Prolonged local contact between the feeding tube and cervical esophageal mucosa may have contributed to repetitive mechanical irritation and pressure-related mucosal injury. Swallowing and cervical motion may have further increased tube–mucosa interaction. The C4-C6 region is located immediately cranial to the thoracic inlet, where relative anatomic confinement may limit esophageal distension and potentially increase focal contact between the feeding tube and esophageal wall. The consistent C4-C6 distribution therefore raises the possibility that local mechanical injury contributed to stricture formation in addition to reflux-related esophagitis. This mechanism remains speculative because mucosal injury was not documented before stricture formation.

Endoscopic balloon dilatation is an established treatment for benign esophageal strictures in dogs and cats. In case 3, 3 balloon dilatation sessions performed at 1-week intervals resulted in enlargement of the esophageal lumen and were followed by sustained clinical improvement, with no recurrent regurgitation at 3 months.

In conclusion, focal cervical esophageal strictures developed after prolonged nasoesophageal tube placement in 3 cats. The strictures were consistently located at the C4-C6 level, and regurgitation developed shortly after tube removal. Prolonged nasoesophageal tube placement may represent a potential risk factor for esophageal stricture formation in cats.

## Supplementary Material

Supplementary_Figure_S1_aalag289

## Off-label antimicrobial declaration

The authors declare no off-label use of antimicrobials.

## Institutional animal care and use committee or other approval declaration

Institutional animal care and use committee approval was not required for this retrospective case series. Informed owner consent was obtained for the use of clinical data and images for publication.

## Abbreviations

DEHP: di(2-ethylhexyl) phthalate
RI: reference interval
